# Supplementary material for: Complex‐centric proteome profiling by SEC‐SWATH‐MS
Source: Mol Syst Biol. 2019 Jan 14;15(1):e8438. doi: 10.15252/msb.20188438 (PMC6346213; doi:10.15252/msb.20188438)
Supplement: Supplementary file 8 — Dataset EV7 [file MSB-15-e8438-s008.zip › feature_plots_string/P15313.pdf]

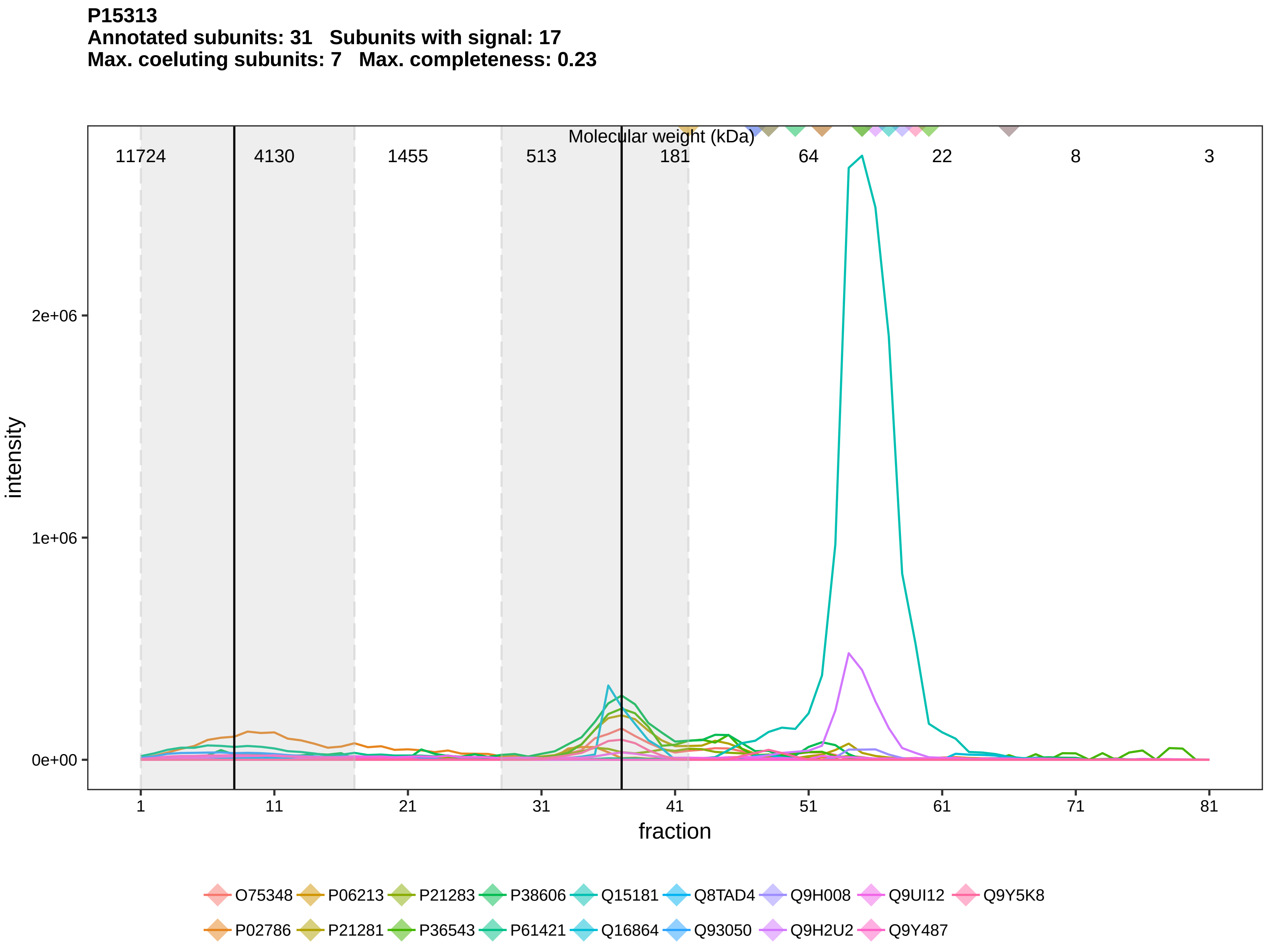
P15313
Annotated subunits: 31 Subunits with signal: 17
Max. coeluting subunits: 7 Max. completeness: 0.23
Molecular weight (kDa)
11724
4130
1455
513
181
64
22
8
3
intensity
2e+06
1e+06
0e+00
fraction
1
11
21
31
41
51
61
71
81
O75348
P06213
P21283
P38606
Q15181
Q8TAD4
Q9H008
Q9UI12
Q9Y5K8
P02786
P21281
P36543
P61421
Q16864
Q93050
Q9H2U2
Q9Y487
